# Matrix Stiffness Promotes DRP1‐Mediated Myofibroblast Senescence to Drive Silica‐Induced Pulmonary Fibrosis

**DOI:** 10.1111/acel.70275

**Published:** 2025-10-17

**Authors:** Xinying Zeng, Jingya Li, Jiaxin Wang, Jiaxin Zhang, Yuhua Wang, Yan Wang, Yifei Wang, Lin Tian, Zhonghui Zhu

**Affiliations:** ^1^ Department of Occupational and Environmental Health, School of Public Health Capital Medical University Beijing China; ^2^ Beijing Key Laboratory of Environment and Aging, School of Public Health Capital Medical University Beijing China; ^3^ Experimental Teaching Center of Public Health and Preventive Medicine, School of Public Health Capital Medical University Beijing China

**Keywords:** DRP1, matrix stiffness, mitochondrial stress, myofibroblast senescence, silicosis

## Abstract

Silicosis is an occupational lung disease characterized by diffuse pulmonary fibrosis resulting from inhalation of silica particles. As the disease progresses, lung tissue stiffness continuously increases, driving persistent activation and accumulation of myofibroblasts. However, whether these cells undergo senescence in response to prolonged high matrix stiffness and how such senescence impacts fibrosis progression remain unclear. Here, we established an in vitro model using decellularized lung matrices with varying stiffness to simulate the fibrotic mechanical microenvironment. We found that increased matrix stiffness upregulated mitochondrial fission protein DRP1, inducing excessive mitochondrial fragmentation and accumulation of mitochondrial reactive oxygen species (mtROS), leading to oxidative stress, DNA damage, and myofibroblast senescence. Treatment with the mitochondria‐targeted antioxidant Mitoquinone mesylate (MitoQ10) effectively alleviated these effects. Moreover, senescent myofibroblast‐derived secretions promoted fibroblast activation and collagen deposition via paracrine signaling, exacerbating fibrotic remodeling. These findings identify matrix stiffness‐driven cellular senescence as a critical mechanism in silicosis progression, providing a rationale for targeting senescent cells as an antifibrotic therapeutic strategy.

## Graphical abstract

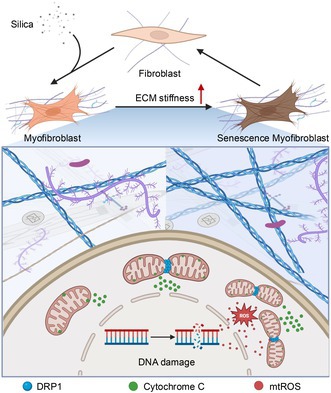

## 1 Introduction

With the rapid advancement of industrialization, occupational lung diseases have become a growing global health concern. Among them, silica‐induced pulmonary fibrosis is particularly severe, characterized by silicotic nodule formation and diffuse fibrosis driven by persistent fibroblast activation and excessive extracellular matrix (ECM) deposition (Baum and Arnold 2025; Sherekar et al. 2025; Wang et al. 2024). Despite a well‐defined etiology, effective therapies remain lacking due to the complex, multicellular nature of the disease.

In recent years, increasing attention has been paid to how the biophysical properties of the tissue microenvironment—particularly matrix stiffness—regulate tissue homeostasis and pathological remodeling. As a fundamental mechanical cue, matrix stiffness profoundly influences key cellular behaviors such as migration, proliferation, differentiation, and metabolism, thereby shaping both physiological processes and disease progression (Sun et al. 2022). In fibrotic diseases, progressive matrix stiffening arises from excessive deposition of ECM proteins—primarily collagen, fibronectin, and hyaluronic acid—by activated fibroblasts (Hupfer et al. 2021). This stiffened matrix, in turn, reinforces fibroblast activation, sustains the myofibroblast phenotype, and perpetuates pathological remodeling through feed‐forward mechanical signaling loops (Tschumperlin et al. 2018). Myofibroblasts, as the principal effector cells in fibrosis, exhibit features of both fibroblasts and smooth muscle cells, including alpha‐smooth muscle actin (α‐SMA) expression, prominent stress fibers, and enhanced contractility (Ju et al. 2024; Jun and Lau 2018). While transient myofibroblast activation is essential for tissue repair, sustained mechanical stress drives their persistent activation and accumulation, leading to excessive ECM deposition, scar formation, and further matrix stiffening (Xu, Li, et al. 2025). More importantly, mechanical cues generated by the stiffened matrix reinforce the profibrotic activity of myofibroblasts via a positive feedback loop, exacerbating fibrosis (Bochaton‐Piallat et al. 2016). Despite this growing recognition, how matrix stiffening drives functional transitions in myofibroblasts—particularly in the context of silica‐induced pulmonary fibrosis—remains poorly understood.

Cellular senescence is an irreversible cell cycle arrest triggered by DNA damage, oncogene activation, oxidative stress, or telomere shortening, primarily mediated by the p53/p21 and p16INK4a/pRb tumor suppressor pathways (Suryadevara et al. 2024). Growing evidence highlights its pivotal role in fibrotic lung diseases (Schafer et al. 2017). Senescent cells contribute to both idiopathic pulmonary fibrosis (IPF) and bleomycin‐induced fibrosis (Guan et al. 2022). Notably, senescent myofibroblasts accumulate in fibrotic lung tissues of pulmonary fibrosis patients (Kato et al. 2020). Similarly, Luo Z et al. reported that eliminating senescent myofibroblasts during peak fibrosis alleviates symptoms and halts progression (Shen et al. 2024), suggesting that myofibroblast senescence is a key driver of fibrosis. Beyond injury‐induced triggers, matrix stiffening has also been implicated in senescence. Shanahan et al. identified ECM stiffening as a hallmark of vascular aging (Faleeva et al. 2024). Likewise, matrix stiffening accelerates chondrocyte and cartilage senescence, promoting osteoarthritis (Fu et al. 2024), and drives epithelial cell senescence in bleomycin‐induced lung fibrosis (Ba et al. 2025). However, whether matrix stiffening induces myofibroblast senescence in silicosis remains largely unexplored. Understanding how matrix stiffening intersects with senescence in silica‐induced pulmonary fibrosis is critical for clarifying their roles in disease pathogenesis.

Mitochondria are central regulators of cellular metabolism, reactive oxygen species (ROS) production, and stress responses, and their dysfunction has been shown to be closely associated with fibrosis and cellular senescence (Huang et al. 2023; Zhang et al. 2025). Dynamin‐related protein 1 (DRP1), a key regulator of mitochondrial fission, plays a critical role in maintaining mitochondrial dynamics and homeostasis. Previous studies have reported that excessive activation of DRP1 is often accompanied by abnormal mitochondrial morphology and accumulation of mtROS, an oxidative stress state that may further impact cellular function and fate (Zeng et al. 2023). Therefore, research focusing on DRP1 and mitochondrial oxidative stress has gradually emerged as an important approach to understanding the pathological processes of cellular senescence and fibrosis.

In this study, we aimed to systematically investigate whether matrix stiffening contributes to the progression of silica‐induced pulmonary fibrosis by promoting myofibroblast senescence. To this end, we established an ex vivo culture model using decellularized lung matrices of varying stiffness and performed functional interventions with the mitochondrial‐targeted antioxidant MitoQ10. Through this approach, we sought to delineate the intrinsic links among matrix stiffness, cellular senescence, and fibrotic progression, and to elucidate the underlying mechanisms. Our findings may provide novel mechanistic insights into the pathogenesis of silicosis and identify potential therapeutic targets for intervening in pulmonary fibrosis.

## 2 Materials and Methods

### 2.1 Animals and Experimental Design

Eighteen male C57BL/6J mice (Charles River, Beijing, China) were randomly assigned to control (*n* = 6), silica 4‐week (*n* = 6), and silica 8‐week (*n* = 6) groups. Mice were maintained under standard conditions with a 12 h light–dark cycle and free access to food and water. Pulmonary fibrosis was induced by intratracheal instillation of silica (5 mg/50 μL/mouse, S5631, Sigma, USA); controls received sterile saline. A single age‐matched saline control group (corresponding to the 4‐week silica group) served as the control for both time points. Mice were euthanized at weeks 4 and 8 post‐instillation, and lung tissues were collected for analysis. All procedures were approved by the Institutional Animal Care and Use Committee of Capital Medical University (AEEI‐2024‐104).

### 2.2 Cell Culture

Primary lung fibroblasts were isolated from C57BL/6J mice (3–7 days old) as previously described (Zhou et al. 2019). Briefly, lung tissues were minced and digested at 37°C for 1 h with collagenase I (1 mg/mL, C8140, Solarbio, Beijing, China) and DNase I (10 μg/mL, D8071, Solarbio, Beijing, China). Cells were cultured in DMEM/F‐12 (C11330500BT, Gibco, USA) with 10% FBS (10091148, Gibco, USA) and 1% penicillin–streptomycin (KGL2303‐100, KeyGEN, Jiangsu, China). NIH/3 T3 cells (Cell Resource Center, CAS, Beijing, China) were maintained in DMEM (PM150210, Procell, Wuhan, China) supplemented with 10% NBCS (16010159, Gibco, USA) and 1% penicillin–streptomycin. For myofibroblast induction, cells were treated with 5 ng/mL TGF‐β1 (100‐21, PeproTech, USA) for 24 h.

### 2.3 Scanning Electron Microscopy (SEM)

Decellularized lung matrices stored at −80°C were removed and subjected to freeze‐drying. The dried samples were mounted onto conductive adhesive tape and imaged using a scanning electron microscope (SEM, Hitachi S‐4800, Hitachi, Japan).

### 2.4 Preparation and Sectioning of Decellularized Lung Matrices

Decellularized lung matrices were prepared from mice in the control, 4‐week silica, and 8‐week silica groups, following established protocols (Xue et al. 2024). The resulting matrices were stored at −80°C until use. Frozen sections (10 μm) were prepared using a cryostat (Leica, Germany). Based on the group of origin, the matrices were categorized as low (control), medium (4‐week silica), or high (8‐week silica) stiffness.

### 2.5 Atomic Force Microscopy (AFM)

Cryosections of decellularized lung matrices were subjected to force mapping using an atomic force microscope (Bioscope Resolve, Bruker, Germany). All measurements were performed at room temperature within 1 h. Sixteen force curves were collected per group, and data were analyzed using NanoScope Analysis software (version x86_v180r1).

### 2.6 Histological Analysis

Lung tissues were fixed in 4% paraformaldehyde, embedded in paraffin, and sectioned at 5 μm thickness. Sections were stained with hematoxylin and eosin (H&E) and Masson's trichrome to evaluate inflammatory infiltration and collagen deposition. Images were acquired using a digital slide scanner (DX150, Hungary).

### 2.7 Matrix Slice Culture System

NIH/3 T3 cells, primary fibroblasts, and myofibroblasts were seeded onto the surface of decellularized lung matrix cryosections. Cells were cultured in DMEM/F12 medium supplemented with 10% fetal bovine serum (FBS) and penicillin–streptomycin (PS), with medium changes every other day.

To investigate the role of mtROS in myofibroblast function, cells were treated with 50 nM MitoQ10 (HY‐100116A, MCE, USA) when they reached 70%–80% confluence. After 2 h of treatment, MitoQ10‐containing medium was removed, and the cells were cultured in fresh medium for an additional 24 h before subsequent analyses.

### 2.8 Conditioned Medium Treatment of Primary Fibroblasts

Myofibroblasts were seeded onto decellularized lung matrix cryosections and cultured in DMEM/F12 medium containing 10% FBS and 1% PS. After 48 h, the supernatant was collected, filtered through a 0.22 μm membrane, and stored at −80°C.

Primary fibroblasts were maintained in DMEM/F12 medium with 10% FBS and 1% PS and treated with the conditioned medium for 24 h.

### 2.9 Immunofluorescence Staining

Cells cultured on decellularized lung matrix cryosections were fixed and permeabilized with 0.3% Triton X‐100 (T8200, Solarbio, Beijing, China) and blocked with 5% goat serum (ZLI‐9021, ZSGB‐BIO, Beijing, China). Samples were incubated overnight at 4°C with primary antibodies against α‐SMA (ab7817, Abcam), Ki67 (ab279653, Abcam), p53 (60283‐2‐Ig, Proteintech, Wuhan, China), p21 (YM8364, ImmunoWay, USA), p16 (sc‐1661, Santa Cruz, USA), DRP1 (F0328, Selleck, USA), cytochrome c (F2534, Selleck, USA), γ‐H2A.X (ab81299, Abcam), and phospho‐Rb (F2220, Selleck, USA). After washing, fluorophore‐conjugated secondary antibodies were applied for 1 h at room temperature. Nuclei were counterstained with DAPI mounting medium (ZLI‐9600, ZSGB‐BIO, Beijing, China). Images were acquired using a Nikon AX confocal microscope (Nikon, Japan).

### 2.10 Measurement of mtROS

Mitochondrial superoxide levels were assessed using MitoSOX Red (M36008, Thermo Fisher, USA). Briefly, after treatment as described above, myofibroblasts were incubated with 5 μM MitoSOX Red at 37°C for 20 min. Cells were then counterstained with 10 μM Hoechst 33342 (R37165, Thermo Fisher, USA) for 10 min. Fluorescence images were acquired using a fluorescence microscope, and signal intensity was quantified using ImageJ version 1.54f software.

### 2.11 In Vivo Lung Imaging in p16‐3MR Mice

B6.Cg‐Tg(Cdkn2a/luc/RFP/TK)1Cmps/J hemizygous mice (p16‐3MR, JAX #012433) were used to detect senescent cells in vivo (Demaria et al. 2014). Mice received D‐galactose (200 mg/kg/day, G7785, Acima, Beijing, China) via intraperitoneal injection or intratracheal silica (5 mg/50 μL/mouse) for 8 weeks. Lung imaging was performed using an IVM‐CMS3 intravital microscopy system (IVIM Technology, South Korea). Mice were anesthetized with tribromoethanol and injected with FITC‐dextran (2 MDa, 25 mg/kg; Sigma, USA) via the tail vein. Ventilation was maintained with RV‐01 (Kent Scientific) at 24–30 mmHg inspiratory pressure, 120–130 breaths/min, and 2 cm H₂O PEEP. Body temperature was controlled at 37°C using the IVM Temp Module (IVIM Technology). A left thoracotomy was performed between the third and fourth ribs. The lung was stabilized with an IVIM lung imaging chamber under 20–30 mmHg negative pressure (NVC 2300a, EYELA). Imaging was conducted using a 25× water‐immersion objective.

### 2.12 Flow Cytometric Identification of Primary Fibroblasts

Primary fibroblasts were digested into single‐cell suspensions. Cell viability was assessed with Fixable Viability Stain 510 (564406, BD Biosciences, USA), and Fc receptors were blocked with Fc Block (553141, BD). Cells were stained with CD31 (561073, BD), CD45 (561037, BD), CD326 (563478, BD), and LYVE1 (12‐0443‐82, Thermo Fisher, USA). Flow cytometry was performed on a BD LSRFortessa, and data were analyzed using FlowJo v10. The gating strategy included exclusion of debris (FSC/SSC) and doublets (FSC‐H/FSC‐A), followed by removal of dead cells (Viability Stain 510^+^). Fibroblasts were defined as CD31^−^CD45^−^CD326^−^LYVE1^−^ within the live single‐cell population.

### 2.13 Fluorescence‐Activated Cell Sorting (FACS) of Senescent Myofibroblasts

p16‐3MR mice were used to establish an 8‐week silica‐induced lung fibrosis model. Lungs were excised following tribromoethanol anesthesia and processed into single‐cell suspensions using the Lung Dissociation Kit (130‐095‐927, Miltenyi Biotec, Germany). Cells were stained on ice for 45 min with CD31 (561814, BD), CD45 (561037, BD), CD326 (563478, BD), LYVE1 (50‐0443‐80, Thermo Fisher, USA), and CD90 (ab25672, Abcam, USA), and viability was assessed using DAPI (564907, BD). Sorting was performed on a FACS Aria SORP (BD), and data were analyzed with FlowJo v10. Gating involved exclusion of debris (FSC‐A/SSC‐A), doublets (FSC‐H/FSC‐A), and DAPI^+^ dead cells. Fibroblasts were defined as CD31^−^CD45^−^CD326^−^LYVE1^−^CD90^+^, and myofibroblasts were defined as CD31^−^CD45^−^CD326^−^LYVE1^−^CD90^−^, with senescent myofibroblasts identified by high p16‐driven mRFP fluorescence (mRFP^+^).

### 2.14 Senescence‐Associated β‐Galactosidase (SA‐β‐Gal) Staining

SA‐β‐gal staining was performed using a commercial kit (C0602, Beyotime, Jiangsu, China) following the manufacturer's instructions. Cells cultured on matrix sections were stained and imaged using a bright‐field microscope (D‐35578, Leica, Germany).

### 2.15 Quantitative Real‐Time PCR (qRT‐PCR)

Total mRNA was reverse transcribed into cDNA using the TransScript First‐Strand cDNA Synthesis SuperMix (AT301, TransGen Biotech, Beijing, China). Quantitative real‐time PCR (qRT‐PCR) was then performed using the PerfectStart Green qPCR SuperMix (AQ601, TransGen Biotech, Beijing, China) on a CFX96 Real‐Time PCR Detection System (Bio‐Rad, USA). Relative gene expression was calculated using the 2^ΔΔCt^ method, where Ct values were normalized to the internal reference gene (GAPDH) and compared to control samples.

### 2.16 Western Blot Analysis

Total protein was extracted using RIPA buffer with protease and phosphatase inhibitors (P0013K, Beyotime, China). Protein concentrations were determined by BCA assay (BCA02, Dingguo, China). Equal amounts of protein were separated by SDS‐PAGE and transferred to PVDF membranes (IPVH00010, Millipore, USA). Membranes were blocked with 5% non‐fat milk in TBST for 1 h at room temperature, then incubated overnight at 4°C with primary antibodies against Collagen I (ab270993, Abcam, USA), α‐SMA (ab7817, Abcam, USA), Fibronectin (YM8309, ImmunoWay, USA), and GAPDH (2118S, CST, USA) as a loading control. After washing, membranes were incubated with HRP‐conjugated secondary antibodies for 1 h at room temperature. Signals were detected using ECL reagents (P10300, NCM, China) and imaged with a Bio‐Rad chemiluminescence system. Band intensities were quantified using ImageJ v1.54f.

### 2.17 Statistical Analysis

All statistical analyses were performed using IBM SPSS Statistics version 24. Two‐way analysis of variance (two‐way ANOVA) was used to evaluate the effects of matrix stiffness (low, medium, high) and treatment conditions (control vs. MitoQ10) on outcome variables. One‐way ANOVA was applied for comparisons among multiple groups, followed by Tukey's post hoc test where appropriate. Data are presented as mean ± standard deviation (SD). Specific statistical tests used are described in the corresponding figure legends. Statistical significance was defined as follows: *p < 0.05; **p < 0.01; ***p < 0.001.

## 3 Results

### 3.1 Matrix Stiffening Occurs During Silica‐Induced Pulmonary Fibrosis

**FIGURE 1:**
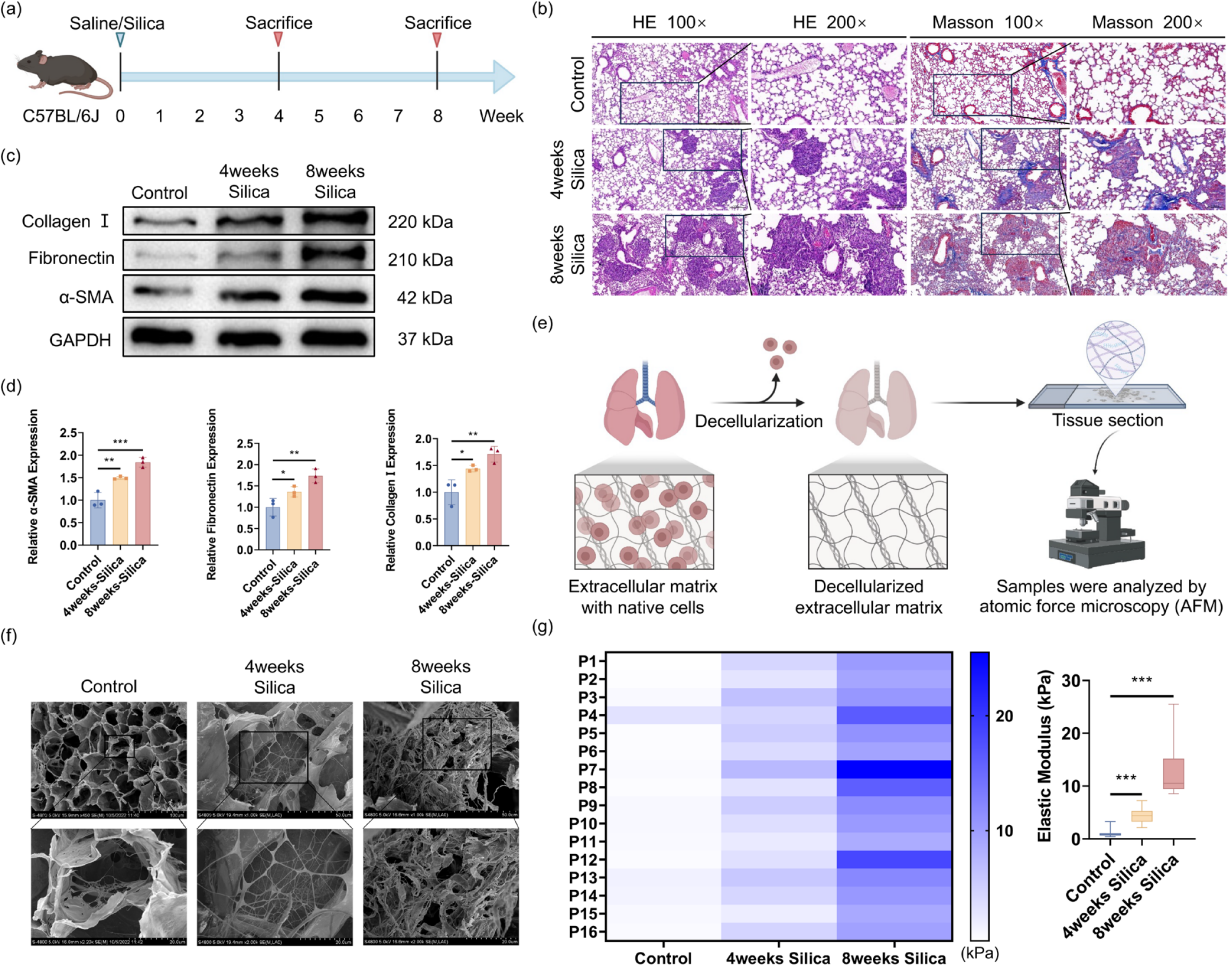
Matrix stiffening occurs during silica‐induced pulmonary fibrosis. (a) Experimental scheme of mouse model. (b) H&E and Masson staining of lung sections showing tissue architecture and collagen deposition (left: 100×, scale bar 200 μm; right: 200×, scale bar 100 μm). (c, d) Western blot and quantification of α‐SMA, Fibronectin, and Collagen I in lung tissue; GAPDH as loading control. Data are presented as mean ± SEM; *p < 0.05, **p < 0.01, ***p < 0.001, *n* = 3. (e) Workflow for decellularized lung matrix preparation and AFM measurement. (f) SEM images of decellularized lung matrix showing preserved 3D structure. (g) AFM‐derived elastic modulus heatmaps (left) and box plots (right) showing local stiffness distribution and overall matrix stiffness in control, silica‐4‐week, and silica‐8‐week groups. Sixteen points (P1—P16) were measured per group. Increased matrix stiffness was observed with prolonged silica exposure. Data are presented as mean ± SEM; ***p < 0.001, *n* = 16.

We established a mouse model with a single intratracheal instillation of silica, followed by observations at 4 and 8 weeks, with saline‐treated mice serving as controls (Figure 1a). HE staining revealed severe alveolar destruction, thickened alveolar septa, and prominent inflammatory cell infiltration in silica‐exposed lungs. Masson's trichrome staining further showed significant collagen deposition in the silica groups, with the 8‐week group exhibiting more extensive fibrosis than the 4‐week group, indicating progressive fibrotic severity over time (Figure 1b). Western blot analysis demonstrated markedly increased protein levels of collagen I, fibronectin, and α‐SMA in the 4‐week and 8‐week silica groups compared with controls, with higher expression in the 8‐week group, confirming progressive fibrosis (Figure 1c,d). Following model validation, we generated structurally intact decellularized lung matrices using pulmonary artery perfusion (Figure 1e). Scanning electron microscopy revealed that control lung matrices exhibited a loose, mesh‐like architecture. In contrast, matrices from silica‐exposed lungs, especially the 8‐week group, displayed increased density, disorganized fiber alignment, and localized collagen aggregation and remodeling, suggesting substantial ECM structural remodeling associated with fibrosis progression (Figure 1f). AFM was then used to measure the elastic modulus of decellularized lung matrices from control, silica 4‐week, and silica 8‐week groups. The results showed a progressive increase in matrix stiffness with prolonged silica exposure. Heatmaps illustrated this trend, with lighter colors in the control group, moderate color intensity in the 4‐week group, and the darkest in the 8‐week group, reflecting increasing stiffness. Box plots further confirmed the distribution of elastic modulus across groups, with statistically significant differences among them (Figure 1g). Based on these measurements, we classified the matrices into low (control), medium (silica 4‐week), and high (silica 8‐week) stiffness groups for subsequent cell functional assays.

Together, these results demonstrate that silica exposure induces progressive pulmonary fibrosis characterized by alveolar destruction, collagen accumulation, upregulation of fibrotic markers, ECM densification, and increased matrix stiffness. The AFM‐derived elastic modulus changes further validate mechanical remodeling of the matrix during fibrosis progression.

### 3.2 High Matrix Stiffness Promotes Fibroblast Proliferation and Activation

**FIGURE 2:**
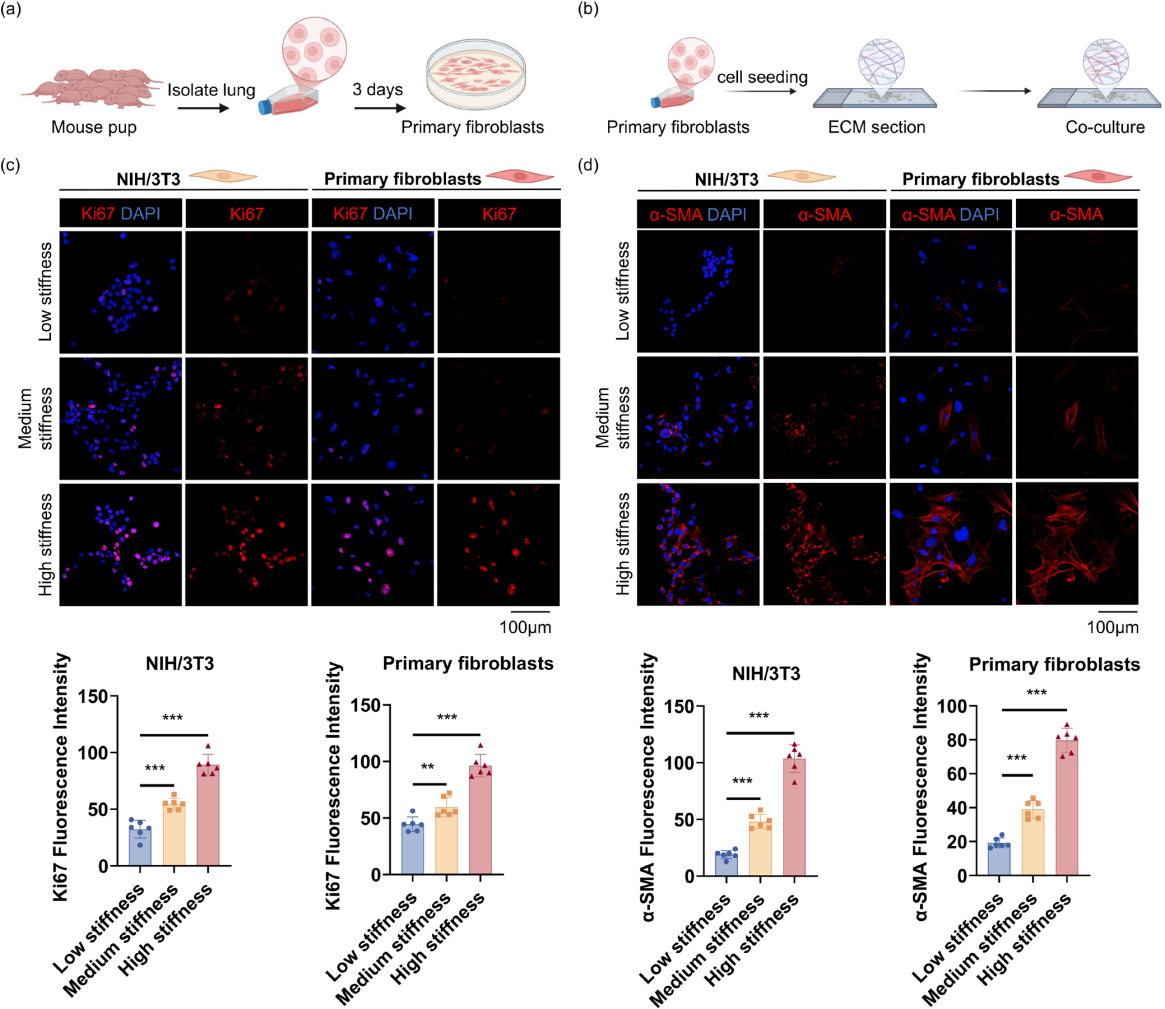
High matrix stiffness promotes fibroblast proliferation and activation. (a) Workflow for isolating primary lung fibroblasts from neonatal mouse lungs. (b) Experimental setup for culturing fibroblasts on decellularized lung matrix slices with different stiffness. (c) Ki67 immunofluorescence staining and quantification of NIH/3 T3 and primary fibroblasts after 48 h of culture on low‐, intermediate‐, or high‐stiffness matrices, showing stiffness‐dependent proliferation (600×, scale bar 100 μm). Data are presented as mean ± SEM; **p < 0.01, ***p < 0.001, *n* = 6. (d) α‐SMA staining and quantification indicating increased fibroblast activation with higher matrix stiffness, particularly in primary cells (600×, scale bar 100 μm). Data are presented as mean ± SEM; ***p < 0.001, *n* = 6.

To investigate how matrix stiffness influences fibroblast behavior, we cultured both NIH/3 T3 cells and primary lung fibroblasts isolated from neonatal mice on decellularized lung matrices with low, medium, and high stiffness (Figure 2a,b). The results showed that fibroblast proliferation increased significantly with matrix stiffness, as evidenced by a higher proportion of Ki67‐positive cells (Figure 2c). Concurrently, fibroblast activation also escalated, with markedly enhanced α‐SMA expression, particularly on high‐stiffness matrices (Figure 2d). Notably, primary lung fibroblasts exhibited more pronounced myofibroblast‐like features under high‐stiffness conditions, including well‐defined stress fibers and intense cytoplasmic accumulation of α‐SMA, indicating a heightened sensitivity to mechanical cues.

Together, these findings demonstrate that increased matrix stiffness significantly promotes fibroblast proliferation and activation. This highlights the critical role of a stiff ECM microenvironment in driving fibroblast functional states, providing important mechanistic insight into how matrix mechanical remodeling regulates cell behavior during pulmonary fibrosis.

### 3.3 High Matrix Stiffness Induces Myofibroblast Senescence

**FIGURE 3:**
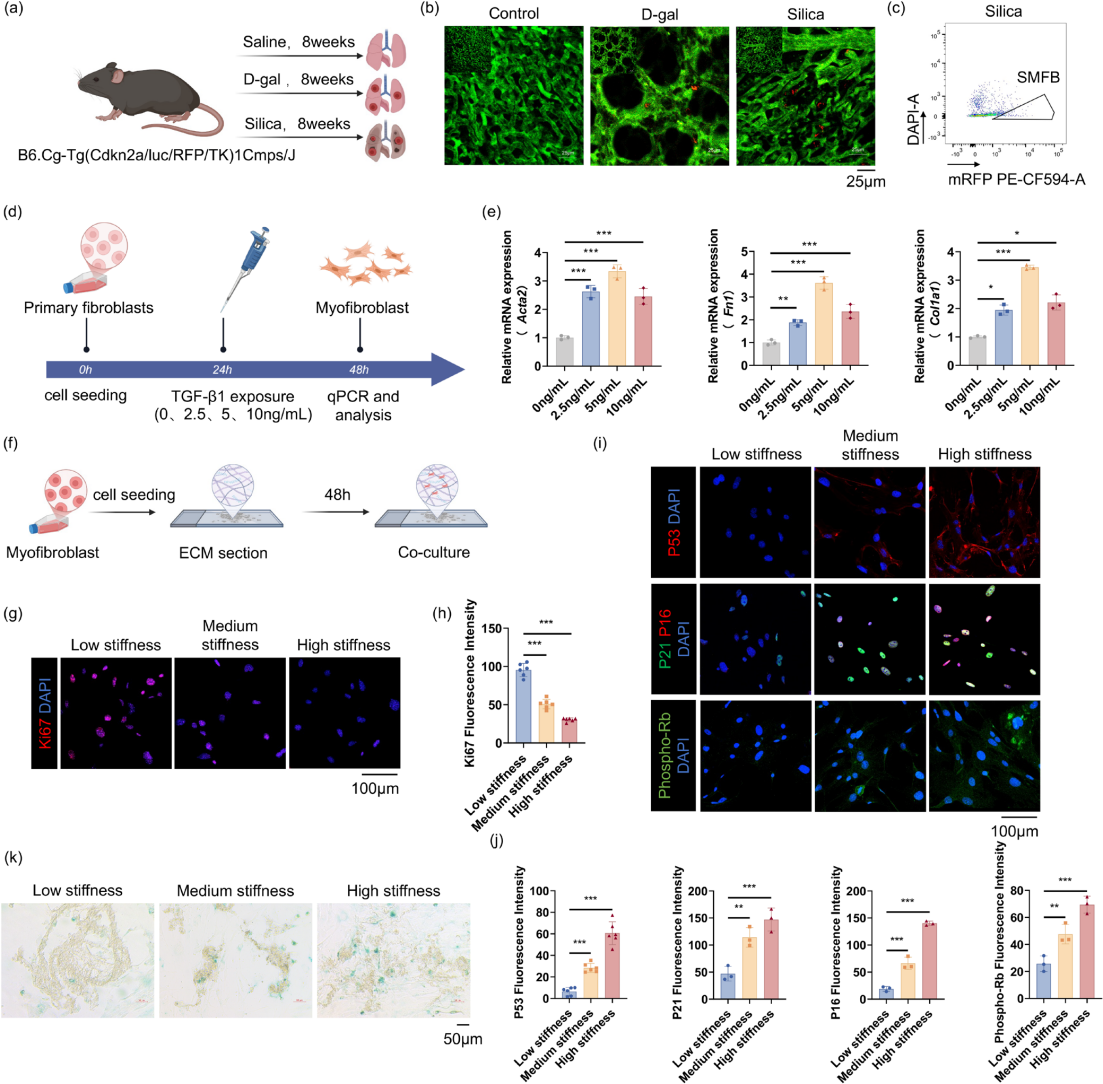
High matrix stiffness induces myofibroblast senescence. (a) Experimental scheme using transgenic mRFP reporter mice treated with saline (control), D‐gal, or silica for 8 weeks. (b) In vivo imaging showing increased red fluorescence in D‐gal and silica groups, indicating senescent cell accumulation; structural disruption observed in the silica group (250×, scale bar 25 μm). (c) Flow cytometry of lung single‐cell suspensions from silica‐exposed mice identified mRFP^+^ senescent myofibroblasts after excluding CD45^+^, CD326^+^, CD31^+^, LYVE1^+^ and CD90^+^ cells. (d) Workflow for in vitro induction of myofibroblasts via TGF‐β1. (e) qPCR validation of *Acta2*, *Col1a1*, and *Fn1* upregulation after TGF‐β1 treatment. Data are presented as mean ± SEM; *p < 0.05, **p < 0.01, ***p < 0.001, *n* = 3. (f) Myofibroblasts were seeded on low‐, medium‐, and high‐stiffness decellularized matrices and cultured for 48 h. (g, h) Ki67 staining and quantification showed reduced proliferation with increasing stiffness (600×, scale bar 100 μm). Data are presented as mean ± SEM; ***p < 0.001, *n* = 6. (i, j) Staining of p53, p21, p16, and phospho‐Rb revealed increased expression on high‐stiffness matrices (600×, scale bar 100 μm). Data are presented as mean ± SEM; **p < 0.01, ***p < 0.001, *n* = 6 for p53, *n* = 3 for p21, p16, and phospho‐Rb. (k) SA‐β‐gal staining confirmed more senescent cells in the high‐stiffness group (200×, scale bar 50 μm).

Building on the finding that high matrix stiffness promotes fibroblast activation, we next investigated whether this mechanical environment also induces myofibroblast senescence. A transgenic senescence‐reporter mouse model expressing mRFP in senescent cells was used for real‐time in vivo monitoring. The experimental groups included a saline control, a D‐galactose‐induced positive control, and an 8‐week silica exposure group (Figure 3a). In vivo imaging revealed significantly elevated mRFP signals in the lungs of both the D‐galactose and silica groups compared to controls, indicating an accumulation of senescent cells in lung tissue (Figure 3b). Notably, the silica group exhibited prominent alveolar structural disruption and tissue remodeling, further supporting the notion that lung senescence and fibrosis occur concurrently. Next, we used the transgenic senescence‐reporter mice with 8‐week silica exposure to identify senescent myofibroblasts in lung tissue via flow cytometry. Single‐cell suspensions were prepared, and a negative selection strategy was applied to exclude immune cells (CD45^+^), epithelial cells (CD326^+^), and endothelial cells (CD31^+^), isolating the CD45^−^CD326^−^CD31^−^CD90^−^ stromal population. mRFP positive cells were detected within this population, indicating that silica exposure induced the formation of senescent myofibroblasts in the lung (Figure 3c). The detailed gating strategy is provided in Figure S4. These results suggest that under conditions of high matrix stiffness and an inflammatory microenvironment, activated myofibroblasts can undergo senescence.

To extend our in vivo findings, we established an in vitro model to mimic the stiffness‐associated matrix microenvironment and assess its impact on myofibroblast senescence.

Primary fibroblasts were first activated into myofibroblasts by TGF‐β1 stimulation, as confirmed by increased expression of *Acta2*, *Col1a1*, and *Fn1* (Figure 3d,e). The myofibroblasts were then cultured on decellularized lung matrices with low, medium, or high stiffness. After 48 h, immunofluorescence revealed a stiffness‐dependent reduction in Ki67‐positive cells, indicating suppressed proliferation (Figure 3f–h). Concurrently, the expression of senescence markers p53, p21, p16, and phospho‐Rb was markedly elevated in the high‐stiffness group (Figure 3i,j). Consistent with these findings, senescence‐associated β‐galactosidase staining showed a significant increase in positive cells under high‐stiffness conditions, confirming the acquisition of a senescent phenotype (Figure 3k).

Collectively, both in vivo and in vitro results demonstrate that a stiffened lung matrix not only promotes fibroblast activation into myofibroblasts but also drives these cells into a senescent state under sustained mechanical stress, resulting in the formation of senescent myofibroblasts (SMFB). This process is likely mediated by stress responses triggered by persistent mechanical tension combined with an inflammatory microenvironment, ultimately leading to cell cycle arrest and the initiation of the senescence program.

### 3.4 Senescent Myofibroblasts Promote Fibroblast Activation

**FIGURE 4:**
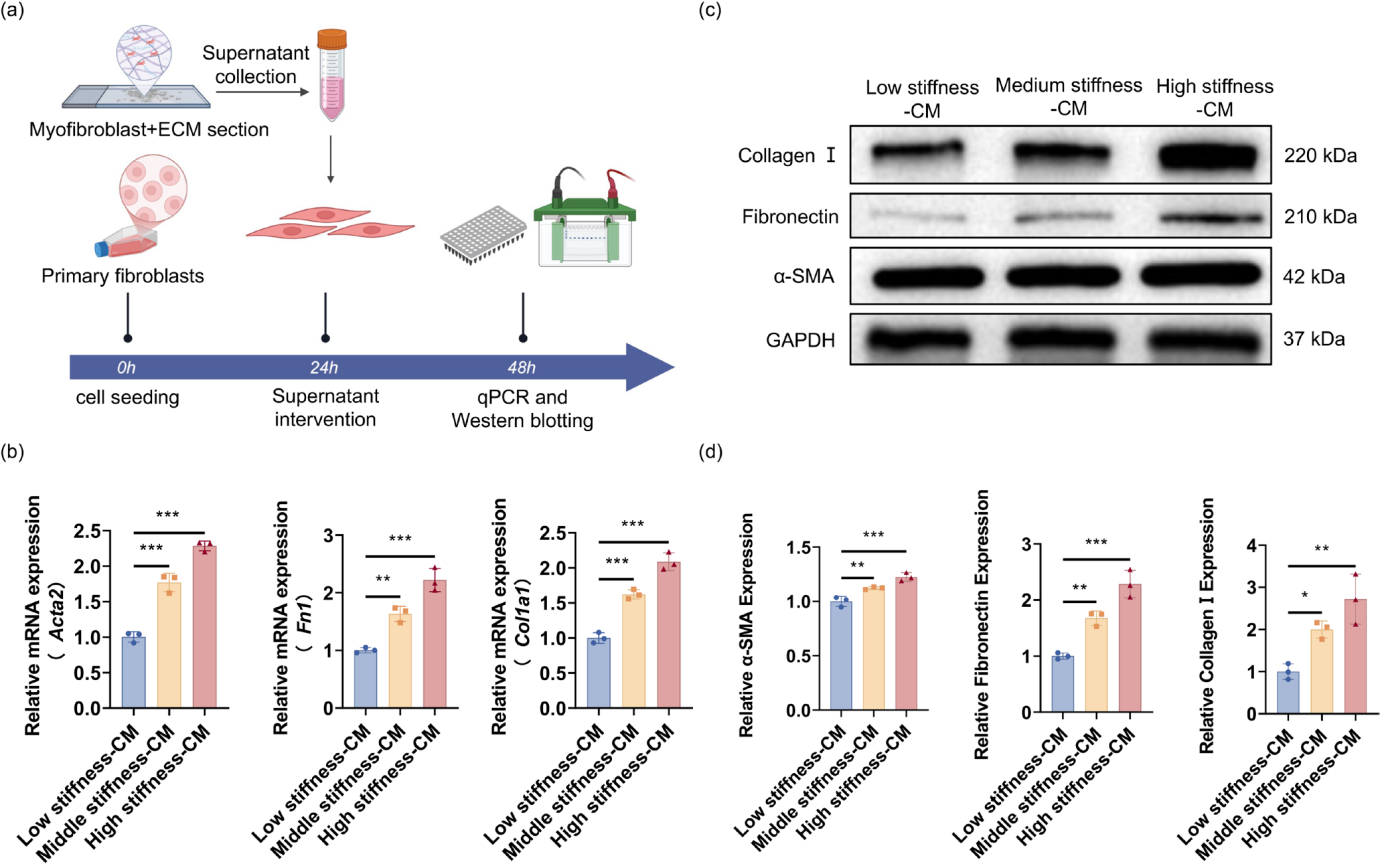
Senescent myofibroblasts promote fibroblast activation. (a) Experimental scheme: TGF‐β1‐induced myofibroblasts were cultured on decellularized matrices with low, medium, or high stiffness for 48 h. Conditioned media (CM) were collected and applied to normal fibroblasts for 24 h to assess activation. (b) qPCR showing stiffness‐dependent increases in *Acta2*, *Col1a1*, and *Fn1* expression in fibroblasts treated with CM. Data are presented as mean ± SEM; **p < 0.01, ***p < 0.001, *n* = 3. (c, d) Western blot and quantification confirming increased α‐SMA, COL1, and FN protein levels in the high‐stiffness CM group. GAPDH as loading control. Data are presented as mean ± SEM; *p < 0.05, **p < 0.01, ***p < 0.001, *n* = 3.

To assess whether senescent myofibroblasts influence neighboring fibroblasts, we collected conditioned media from myofibroblasts cultured for 48 h on matrices with low, medium, or high stiffness and applied it to naïve fibroblasts for 24 h (Figure 4a). Conditioned media from higher‐stiffness matrices induced a stiffness‐dependent increase in fibroblast activation. qPCR analysis showed significant upregulation of *Acta2*, *Col1a1*, and *Fn1* mRNA in fibroblasts treated with media from the high‐stiffness group (Figure 4b). Western blot analysis further confirmed elevated protein levels of α‐SMA, COL1, and FN, with the most pronounced expression under high‐stiffness conditions (Figure 4c,d). These findings indicate that senescent myofibroblasts induced by matrix stiffening secrete pro‐fibrotic factors that enhance fibroblast activation via paracrine signaling, potentially driving a feed‐forward loop that accelerates lung fibrosis progression.

### 3.5 High Matrix Stiffness Induces mtROS Production and DNA Damage via DRP1 Upregulation

**FIGURE 5:**
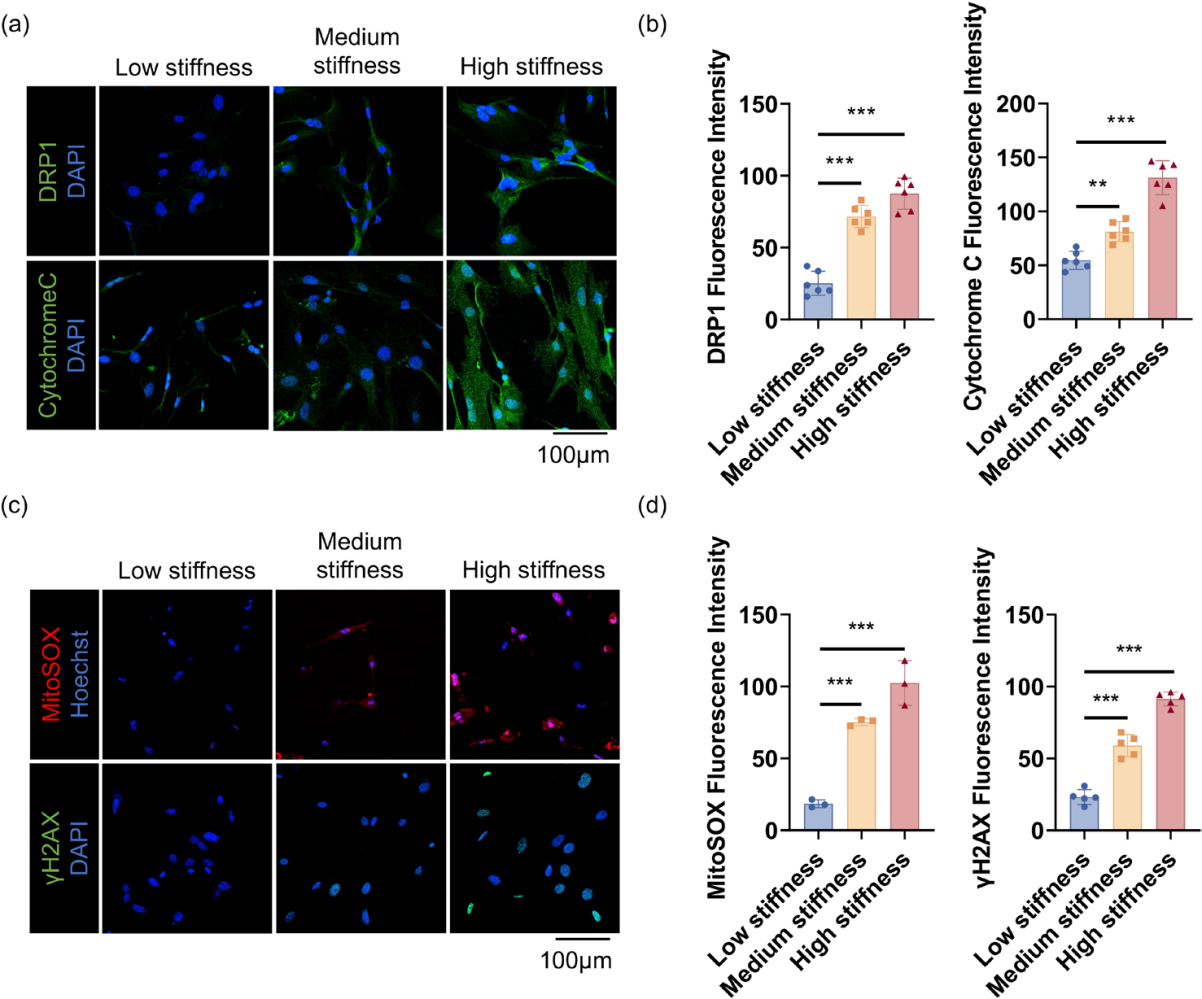
High‐stiffness matrix promotes mitochondrial fission and oxidative stress, leading to DNA damage in myofibroblasts. (a, b) Drp1 and Cytochrome c staining and quantification showing increased Drp1 expression and cytoplasmic Cytochrome c release in myofibroblasts cultured on high‐stiffness matrices (600×, scale bar 100 μm). Data are presented as mean ± SEM; **p < 0.01, ***p < 0.001, *n* = 3. (c, d) MitoSOX and γ‐H2AX staining showing elevated mtROS and aggravated DNA damage in the high‐stiffness group (600×, scale bar 100 μm). Data are presented as mean ± SEM; ***p < 0.001, *n* = 3 for MitoSOX, *n* = 5 for γ‐H2AX.

Given that matrix stiffening promotes myofibroblast senescence, we next sought to investigate whether this mechanical cue also triggers mitochondrial dysfunction—a key driver of cellular stress and senescence. To this end, myofibroblasts were cultured on decellularized lung matrices with low, medium, or high stiffness for 48 h. Higher matrix stiffness triggered pronounced mitochondrial dysfunction, characterized by a marked upregulation of dynamin‐related protein 1 (DRP1), a key mediator of mitochondrial fission. This increase in DRP1 was accompanied by compromised mitochondrial membrane integrity and cytochrome c release (Figure 5a,b), indicating mitochondrial membrane permeabilization and activation of mitochondrial stress pathways. Consistent with these findings, MitoSOX staining revealed a significant rise in mtROS under high‐stiffness conditions, alongside increased γ‐H2AX foci, confirming elevated DNA damage (Figure 5c,d). Collectively, these results demonstrate that matrix stiffening drives DRP1‐mediated mitochondrial fragmentation and oxidative stress, culminating in mtROS accumulation and DNA damage. This mechanistic pathway highlights how mechanical stress perturbs mitochondrial homeostasis and activates intracellular damage responses, fostering a persistent senescent phenotype in myofibroblasts that may perpetuate fibrotic progression.

### 3.6 MitoQ10 Mitigates Matrix Stiffness‐Induced Myofibroblast Senescence

**FIGURE 6:**
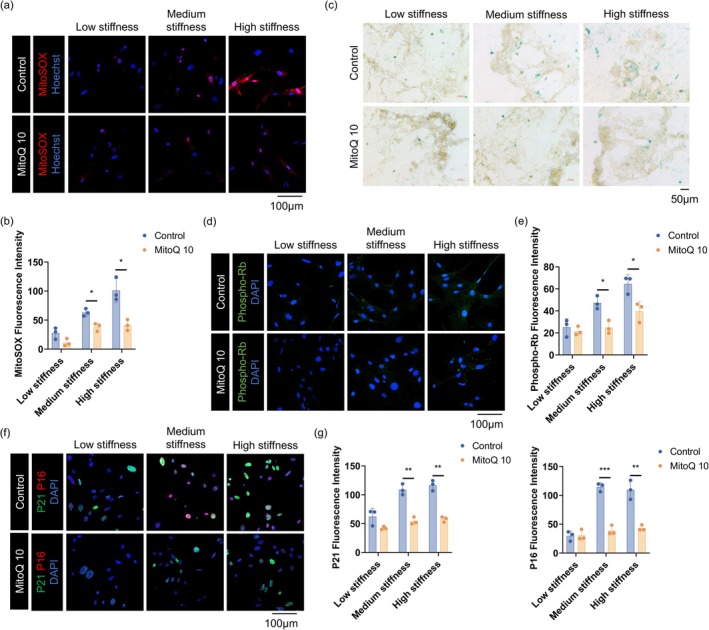
MitoQ10 mitigates matrix stiffness‐induced myofibroblast senescence. (a, b) MitoSOX staining and quantification showing increased mtROS in the high‐stiffness group, reduced by MitoQ10 (600×, scale bar 100 μm). Data are presented as mean ± SEM; *p < 0.05, *n* = 3. (c) SA‐β‐gal staining showing increased senescent cells in the high‐stiffness group, alleviated by MitoQ10 (200×, scale bar 50 μm). (d–g) Staining and quantification of phospho‐Rb, p21, and p16 showing stiffness‐induced upregulation, reversed by MitoQ10 (600×, scale bar 100 μm). Data are presented as mean ± SEM; *p < 0.05, **p < 0.01, ***p < 0.001, *n* = 3.

Building on the observation that mitochondrial oxidative stress mediates matrix stiffness‐induced myofibroblast senescence, we next tested whether the mitochondria‐targeted antioxidant MitoQ10 could rescue this phenotype. TGF‐β1‐activated myofibroblasts were cultured on decellularized lung matrices with low, medium, or high stiffness, with or without MitoQ10 intervention. This treatment markedly reduced mitochondrial oxidative stress under high‐stiffness conditions, as indicated by diminished MitoSOX fluorescence (Figure 6a,b). Correspondingly, senescence‐associated β‐galactosidase staining showed a substantial reduction in blue‐stained positive cells (Figure 6c). In addition, the elevated expression of p21, p16, and phospho‐Rb observed under high stiffness was significantly suppressed by MitoQ10 (Figure 6d–g). These findings demonstrate that scavenging mtROS effectively attenuates matrix stiffness‐induced myofibroblast senescence. This highlights mitochondrial oxidative stress as a critical mediator linking mechanical stress to cellular senescence, offering a potential intervention strategy for fibrosis associated with mechanical microenvironments.

## 4 Discussion

Previous studies have mainly focused on the role of matrix stiffness in promoting fibroblast activation and maintaining cell phenotype, but direct evidence for its ability to induce cellular senescence and the contribution of senescent cells to fibrotic progression has been lacking. In this study, by integrating a decellularized lung matrix–based in vitro culture model with in vivo analyses using senescence‐reporter mice, we provide the first systematic evidence that pathological matrix stiffening drives myofibroblast senescence through DRP1‐mediated mitochondrial dysfunction. In this study, high stiffness markedly upregulates DRP1, leading to excessive mitochondrial fission, cytochrome *c* release, and mtROS accumulation. This triggers oxidative stress, DNA damage, and cell cycle arrest, culminating in a senescent phenotype characterized by increased p16, p21, phosphorylated Rb, and SA‐β‐gal positivity. Treatment with the mitochondria‐targeted antioxidant MitoQ10 attenuated mtROS and reversed senescence, underscoring mitochondrial dysfunction as a critical mechanistic link between ECM stiffening and myofibroblast aging. Moreover, we demonstrate that these senescent myofibroblasts exacerbate fibrosis via paracrine signaling, thereby offering new mechanobiological insights into the progression of silicosis.

Traditionally, silicosis is characterized by persistent and irreversible pulmonary fibrosis (Leung et al. 2012). During disease progression, inhaled silica particles are engulfed by alveolar macrophages, which trigger the release of pro‐inflammatory cytokines and activate fibroblasts. This leads to enhanced fibroblast proliferation, migration, and excessive ECM deposition, ultimately accelerating fibrotic progression (Wang et al. 2023). As a dynamic and complex structural network, the ECM plays a pivotal role in maintaining tissue architecture and homeostasis, while its pathological remodeling is intimately associated with various diseases, including fibrosis and cancer.

A growing body of evidence indicates that progressive ECM accumulation—particularly of collagen—significantly increases matrix stiffness during fibrosis (Loomis et al. 2022). Consistent with our findings, He et al. demonstrated that mechanical stress directly promotes fibroblast activation and myofibroblast differentiation. As the primary effector cells in pulmonary fibrosis, myofibroblasts not only contribute to excessive ECM production but also dynamically respond to mechanical signals within the fibrotic microenvironment (He et al. 2024). Through their contractile forces and remodeling capabilities, myofibroblasts further exacerbate local ECM stiffening, creating a self‐reinforcing fibrotic microenvironment. ECM stiffening is a hallmark of tissue aging. It disrupts mechanotransduction and alters cellular responses to mechanical cues, thereby worsening aging‐related pathologies (Han et al. 2025). Supporting this concept, our study demonstrates that ECM stiffness progressively increases in a silica‐induced murine model of silicosis. Importantly, the stiffened matrix promotes fibroblast‐to‐myofibroblast transition and drives myofibroblast senescence. Collectively, these findings underscore the pivotal role of ECM mechanical properties in coupling fibrotic progression with cellular fate determination, contributing to a mechanotransduction‐driven pathogenic loop within the fibrotic niche.

The free radical theory of aging posits that excessive ROS contribute to cellular senescence (Harman 1956). Among these, mtROS are major drivers of oxidative stress and mitochondrial dysfunction. However, the mechanistic role of mitochondrial dynamics, particularly DRP1‐mediated fission, in senescence remains underexplored (Xu, Pang, et al. 2025). Emerging evidence indicates that mitochondrial fission is hyperactivated under some senescence‐inducing conditions (Zhang et al. 2023). DRP1, the master regulator of mitochondrial fission, orchestrates this process. High matrix stiffness has been shown to activate DRP1, driving mitochondrial fragmentation and oxidative stress (Ke et al. 2023). Similarly, lactate metabolism upregulates DRP1 in bleomycin‐induced fibrosis, promoting mtROS accumulation, which can be mitigated by DRP1 inhibition (Sun et al. 2024). Excessive mtROS damages telomeric DNA, activates DNA damage responses, and accelerates senescence, thereby contributing to fibrosis progression (Li, Kang, et al. 2024). MitoQ10, a mitochondria‐targeted antioxidant, shows therapeutic potential across multiple mitochondrial dysfunction‐related pathologies. Our study confirms that MitoQ10 reduces mtROS and mitigates myofibroblast senescence in high‐stiffness conditions. We further observed increased cytochrome c release under matrix stiffening, consistent with DRP1‐driven mitochondrial permeabilization (Pedrera et al. 2025). Beyond apoptosis, sublethal mitochondrial permeabilization (miMOMP) facilitates the cytosolic release of mitochondrial DNA, triggering SASP activation and reinforcing the senescence phenotype (Victorelli et al. 2023). Collectively, our findings show that matrix stiffness drives DRP1‐dependent mitochondrial fragmentation and increases mtROS and cytochrome c release, leading to myofibroblast senescence. This highlights mitochondrial dynamics as a key mechanobiological transducer linking ECM stiffening to senescence‐driven fibrosis progression.

Cellular senescence causes intrinsic functional decline. It also shapes the microenvironment through secretion of inflammatory and bioactive factors, thereby contributing to the initiation and progression of pulmonary fibrosis (Farhat et al. 2025). A hallmark of senescent cells is the senescence‐associated secretory phenotype (SASP), which encompasses pro‐inflammatory cytokines, chemokines, proteases, and growth factors. These secreted factors influence neighboring cells via paracrine signaling, driving secondary senescence or activating fibroblasts and other stromal cells, thereby sustaining chronic inflammation and promoting fibrotic matrix remodeling (Grootaert 2024). For instance, senescent lung epithelial cells have been shown to promote fibroblast activation and collagen production (Huo et al. 2025). Specifically, senescent alveolar type II (AT2) cells exhibit SASP expression that drives fibroblast proliferation and accelerates fibrosis (Zhou et al. 2022). In chronic fibrotic conditions, this senescence‐driven paracrine network likely serves as a key mechanistic link connecting mechanical stress, cellular stress responses, and tissue structural disruption. Supporting this concept, Martin‐Vicente et al. (2025) demonstrated that mechanical stretch induces lung epithelial cell senescence, which in turn enhances fibroblast activation. Consistent with these findings, our study reveals that conditioned media from stiff matrix‐induced senescent myofibroblasts promotes fibroblast activation and collagen synthesis. Although the precise SASP composition remains to be characterized, this observation suggests that senescent myofibroblasts contribute to the fibrotic microenvironment through paracrine signaling, further amplifying disease progression. This finding establishes a basis for future investigations into the paracrine role of myofibroblast senescence in fibrosis. Targeting senescent cells has thus emerged as a promising antifibrotic strategy. Recent studies highlight senolytics as potential tools to eliminate or modulate senescent cells and suppress SASP‐driven signaling to mitigate fibrosis (Li, Chen, et al. 2024). These insights offer new therapeutic opportunities for silicosis and fibrosis management. Further elucidation of the SASP profile and regulatory mechanisms in senescent myofibroblasts will be instrumental in advancing targeted antifibrotic interventions.

A key strength of this study is the establishment of an in vitro model that replicates the mechanical properties of fibrotic tissue. By culturing primary myofibroblasts on decellularized lung matrices with defined stiffness, we successfully simulated the pathological ECM mechanical environment and its influence on cell fate. This approach enhances physiological relevance and provides a robust platform for exploring how mechanical stress drives mitochondrial dysfunction and senescence. We also acknowledge areas for further investigation that could strengthen our findings. While our results support DRP1 upregulation as a central mediator of stiffness‐induced senescence, we did not extend the investigation to in vivo models with genetic manipulation of DRP1. This limits the ability to fully establish causality in the context of tissue fibrosis. Additionally, our study focused on the DRP1‐mitochondrial fission pathway but did not explore other mechanotransduction pathways such as YAP/TAZ or integrin‐FAK, which may also play crucial roles. Moreover, we primarily investigated ECM stiffness, but did not address changes in ECM composition that could interact with mechanical cues to influence cell behavior. Future studies incorporating in vivo genetic models and proteomic profiling will be crucial for delineating how ECM mechanical and biochemical properties collectively shape myofibroblast behavior in fibrotic progression. This will contribute to a more comprehensive understanding of fibrosis mechanisms and aid the development of targeted therapeutic strategies.

## 5 Conclusion

This study reveals that matrix stiffness drives silicosis‐associated fibrosis through mtROS‐dependent myofibroblast senescence. A stiffened ECM upregulates DRP1, promotes mitochondrial fission, and induces cytochrome c release and mtROS accumulation, leading to DNA damage and cellular senescence. Senescent myofibroblasts further exacerbate fibrosis by activating neighboring fibroblasts via paracrine signaling. Notably, the mitochondria‐targeted antioxidant MitoQ10 effectively reduces oxidative stress and senescence, highlighting its therapeutic potential. Together, these findings establish a mechanistic link between matrix stiffness, mitochondrial dysfunction, and senescence‐driven fibrosis. This work provides a conceptual framework for targeting senescent myofibroblasts as an antifibrotic strategy in silicosis and related fibrotic diseases.

## Author Contributions

**Xinying Zeng:** experimental operation, data analyses. **Jingya Li:** conceptualization, data analyses, material acquisition, original draft preparation. **Jiaxin Wang:** conceptualization, data analyses, material acquisition, original draft preparation; **Jiaxin Zhang:** methodology, investigation. **Yuhua Wang:** methodology, investigation. **Yan Wang:** technical consulting, writing, review and editing, supervision. **Yifei Wang:** experimental verification, data analyses. **Lin Tian:** conceptualization, writing, review and editing, supervision. **Zhonghui Zhu:** conceptualization, writing, supervision, and funding acquisition.

## Ethics Statement

This work has received approval for research ethics from the Animal Experiments and Experimental Animal Welfare Committee of Capital Medical University, and a proof/certificate of approval is available upon request.

## Conflicts of Interest

The authors declare no conflicts of interest.

## Supporting information


**Figure S1:** Flow cytometric identification of primary fibroblasts.
**Figure S2:** Quantification of mtROS levels after treatment with different concentrations of MitoQ10.
**Figure S3:** MitoQ10 reduces DRP1 expression in myofibroblasts cultured on stiff matrices.
**Figure S4:** Flow cytometric sorting of senescent myofibroblasts from silica‐treated p16‐3MR mice.

## Data Availability

The data that support the findings of this study are available from the corresponding author upon reasonable request.
