# Supplementary material for: Matrix Stiffness Promotes DRP1‐Mediated Myofibroblast Senescence to Drive Silica‐Induced Pulmonary Fibrosis
Source: Aging Cell. 2025 Oct 17;24(12):e70275. doi: 10.1111/acel.70275 (PMC12686568; doi:10.1111/acel.70275)
Supplement: Supplementary file 1 — Figure S1: Flow cytometric identification of primary fibroblasts. Figure S2: Quantification of mtROS levels after treatment with different concentrations of MitoQ10. Figure S3: MitoQ10 reduces DRP1 expression in myofibroblasts cultured on stiff matrices. Figure S4: Flow cytometric sorting of senescent myofibroblasts from silica‐treated p16‐3MR mice. [file ACEL-24-e70275-s001.docx]

FigureS1


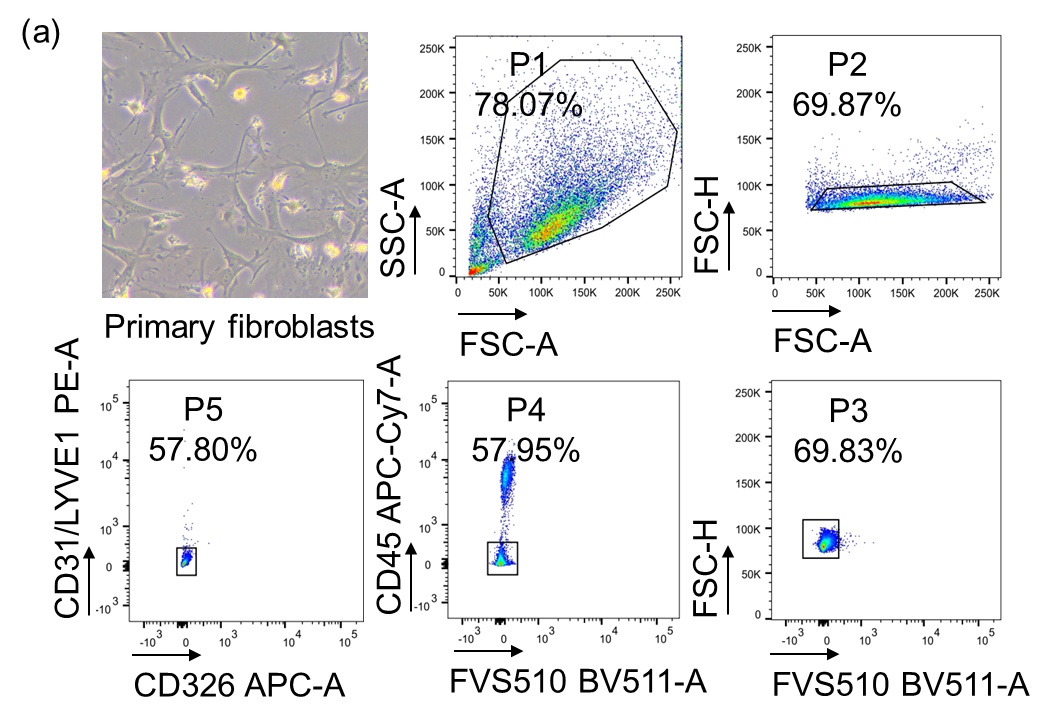


**Figure S1. Flow cytometric identification of primary fibroblasts.**

Representative gating strategy for the identification of primary lung fibroblasts. Debris was excluded by FSC/SSC gating, and singlets were selected by FSC-H versus FSC-A. Live cells were identified by excluding Fixable Viability Stain 510⁺ events. Non-fibroblast lineages including CD31⁺ endothelial cells, CD45⁺ hematopoietic cells, CD326⁺ epithelial cells, and LYVE1⁺ lymphatic endothelial cells were gated out. The fibroblast population was defined as CD31⁻CD45⁻CD326⁻LYVE1⁻ live single cells.

FigureS2


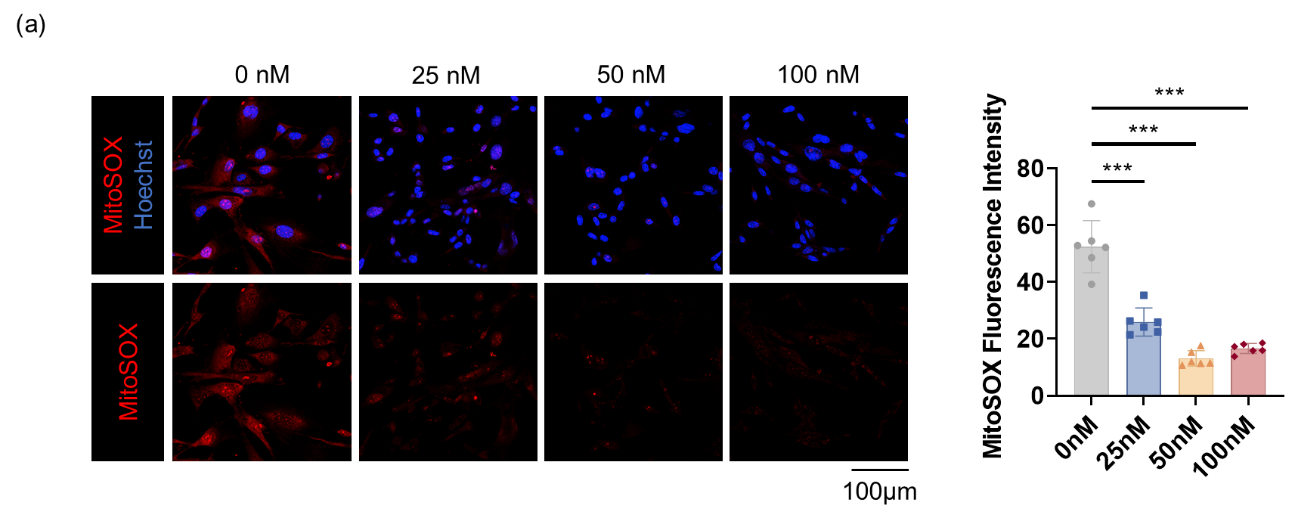


**Figure S2. Quantification of mtROS levels after treatment with different concentrations of MitoQ10.**

MitoSOX fluorescence was measured in myofibroblasts treated with 0 nM, 25 nM, 50 nM, or 100 nM MitoQ10. Representative fluorescence images (left) and statistical analysis of fluorescence intensity (right) are shown. The results indicate that 50 nM MitoQ10 most effectively reduced mtROS levels, and this concentration was selected for subsequent experiments (600×, scale bar 100 μm). Data are presented as mean ± SEM; ****p* < 0.001, n = 6.

FigureS3

**
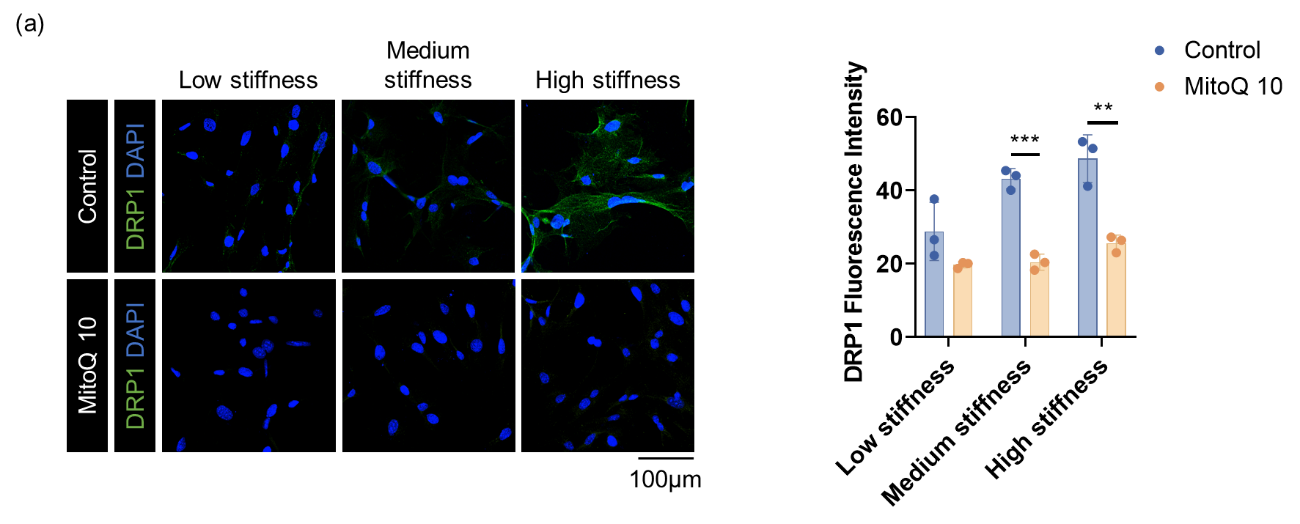
**

**Figure S3. MitoQ10 reduces DRP1 expression in myofibroblasts cultured on stiff matrices.**

Representative immunofluorescence staining and quantification of DRP1 in myofibroblasts cultured on low-, medium-, and high-stiffness matrices, showing that MitoQ10 significantly reduced DRP1 expression on medium- and high-stiffness matrices (600×, scale bar 100 μm). Data are presented as mean ± SEM; ***p* < 0.01, ****p* < 0.001, n = 3.

FigureS4


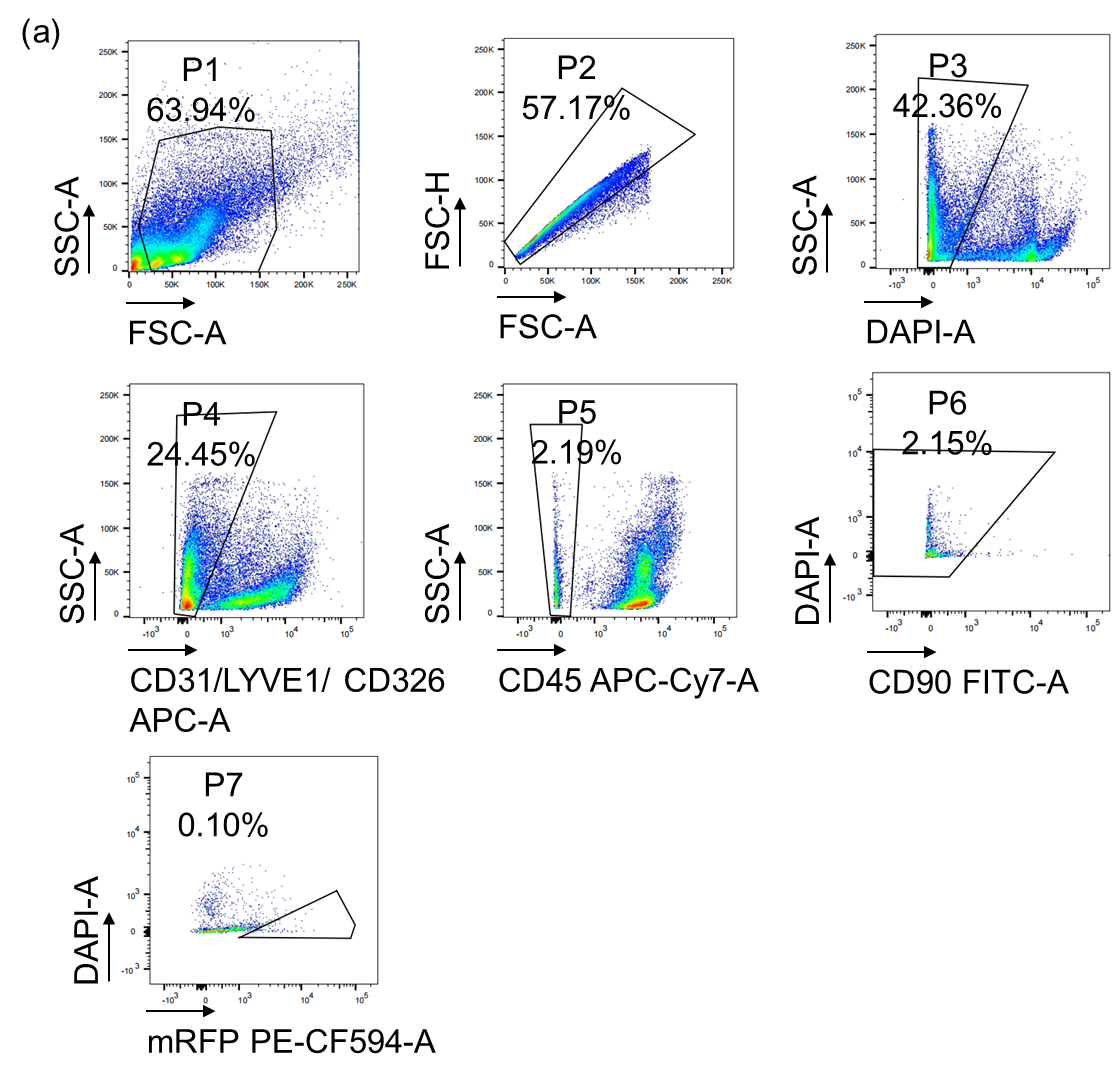


**Figure S4. Flow cytometric sorting of senescent myofibroblasts from silica-treated p16-3MR mice.**

Representative gating strategy for the isolation of senescent myofibroblasts from 8-week silica-treated p16-3MR mice. Debris was excluded by FSC/SSC gating, and singlets were selected by FSC-H versus FSC-A. Live cells were identified by excluding DAPI⁺ events. Non-fibroblast lineages including CD31⁺ endothelial cells, CD45⁺ hematopoietic cells, CD326⁺ epithelial cells, and LYVE1⁺ lymphatic endothelial cells were gated out. Myofibroblasts were defined as CD31⁻CD45⁻CD326⁻LYVE1⁻CD90⁻ cells. Within this myofibroblast population, senescent myofibroblasts were identified by endogenous mRFP fluorescence from the p16-3MR reporter, with mRFP⁺ cells representing senescent myofibroblasts.
